# Comparison of a novel, endoscopic chest tube insertion technique versus the standard, open technique performed by novice users in a human cadaver model: a randomized, crossover, assessor-blinded study

**DOI:** 10.1186/s13049-018-0574-2

**Published:** 2018-12-27

**Authors:** Byron C. Drumheller, Anthony Basel, Sakib Adnan, Joseph Rabin, Jason D. Pasley, Jason Brocker, Samuel M. Galvagno

**Affiliations:** 1 Program in Trauma, Department of Surgery, R Adams Cowley Shock Trauma Center, University of Maryland School of Medicine, 22 South Greene Street, Baltimore, MD 21201 USA; 2 Division of Critical Care Medicine, Department of Medicine, University of Maryland School of Medicine, 22 South Greene Street, Baltimore, MD 21201 USA; 3 School of Medicine, University of Maryland School of Medicine, 22 South Greene Street, Baltimore, MD 21201 USA; 4 Division of Critical Care Medicine, Department of Anesthesiology, University of Maryland School of Medicine, 22 South Greene Street, Baltimore, MD 21201 USA; 5 United States Air Force Center for Trauma and Readiness Sustainment (CSTARS)-Baltimore, University of Maryland School of Medicine, 22 South Greene Street, Baltimore, MD 21201 USA

**Keywords:** Chest tubes, Trauma, Tube thoracostomy, Human cadaver, Emergency care

## Abstract

**Background:**

The technique of tube thoracostomy has been standardized for years without significant updates. Alternative procedural methods may be beneficial in certain prehospital and inpatient environments with limited resources. We sought to compare the efficacy of chest tube insertion using a novel, endoscopic device (The Reactor™) to standard, open tube thoracostomy.

**Methods:**

Novice users were randomly assigned to pre-specified sequences of six chest tube insertions performed on a human cadaver model in a crossover design, alternating between the Reactor™ and standard technique. All subjects received standardized training in both procedures prior to randomization. Insertion site, which was randomly assigned within each cadaver’s hemithorax, was marked by the investigators; study techniques began with skin incision and ended with tube insertion. Adequacy of tube placement (intrapleural, unkinked, not in fissure) and incision length were recorded by investigators blinded to procedural technique. Insertion time and user-rated difficulty were documented in an unblinded fashion. After completing the study, participants rated various aspects of use of the Reactor™ compared to the standard technique in a survey evaluation.

**Results:**

Sixteen subjects were enrolled (7 medical students, 9 paramedics) and performed 92 chest tube insertions (*n* = 46 Reactor™, *n* = 46 standard). The Reactor™ was associated with less frequent appropriate tube positioning (41.3% vs. 73.9%, *P* = 0.0029), a faster median insertion time (47.3 s, interquartile range 38–63.1 vs. 76.9 s, interquartile range 55.3–106.9, *P* < 0.0001) and shorter median incision length (28 mm, interquartile range 23–30 vs. 32 mm, interquartile range 26–40, *P* = 0.0034) compared to the standard technique. Using a 10-point Likert scale (1-easiest, 10-hardest) participants rated the ease of use of the Reactor™ no different from the standard method (3.8 ± 1.9 vs. 4.7 ± 1.9, *P* = 0.024). The Reactor™ received generally favorable scores for all parameters on the post-participation survey.

**Conclusions:**

In this randomized, assessor-blinded, crossover human cadaver study, chest tube insertion using the Reactor™ device resulted in faster insertion time and shorter incision length, but less frequent appropriate tube placement compared with the standard technique. Additional studies are needed to evaluate the efficacy, safety and potential advantages of this novel device.

## Introduction

The technique of tube thoracostomy has changed little since it became standard of care for the treatment of traumatic hemopneumothorax in the 1960s [1, 2]. Recent advances in minimally-invasive surgical technology and increasing awareness of complications associated with the traditional technique have led to the development of novel adaptations to the standard steps of chest tube insertion [3–7]. These new methods generally involve changes to the manner of chest wall dissection, using a laparoscopic-type trocar, or tube insertion, using a video-guided endoscopic stylet. While these sophisticated technologies may be useful in unique scenarios, they have limited applicability to many chest tube procedures, specifically those in challenging environments performed by users without extensive experience [8–10]. A practical, safe, compact and easy-to-learn novel tube thoracostomy technique can have widespread relevance across many arenas, from hospital insertion to austere pre-hospital conditions, such as the deployed environment. Furthermore, such a device would have utility for non-surgeons working in intensive care units where tube thoracostomy may be less frequently performed.

**Fig. 1:**
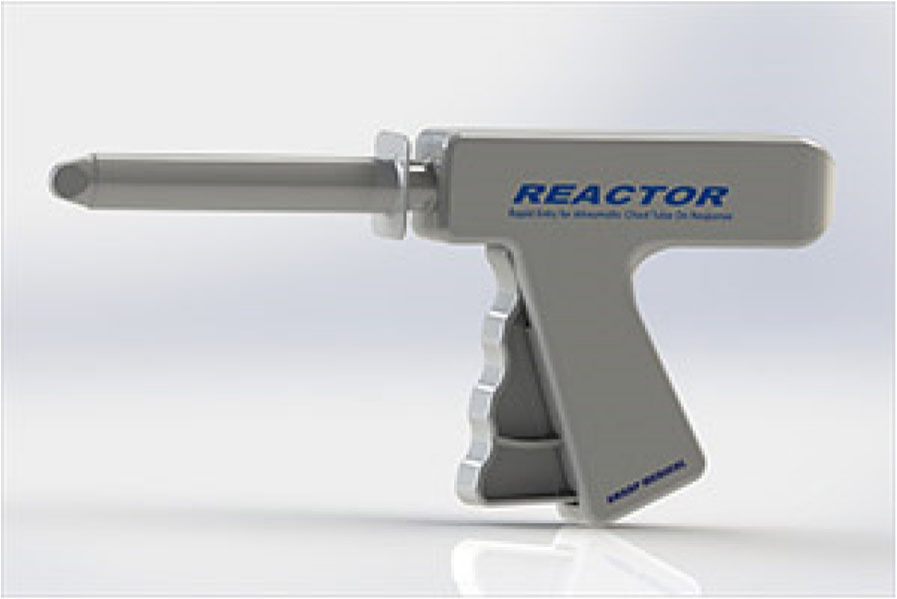
The Reactor™ Endoscopic Chest Tube Device (reprinted with permission from Sharp Medical Products, LLC)

The Reactor™ (Sharp Medical Products, LLC., Geneva, Il) is an FDA-approved, newly designed, hand-held device created for the purposes of minimally-invasive chest tube insertion (Fig. 1) [11]. The device consists of an elliptically shaped probe with an eccentric retractable blade at the distal end and a sheath mounted over the proximal portion. Activated by a trigger mechanism, the blade advances and rotates with exposure of a 1 mm cutting edge, allowing for sharp dissection, and then rapidly and completely retracts upon release of the trigger. Pressure from the user allows for blunt dissection with the probe if the trigger is not depressed. After entrance into the pleural space, a sheath is advanced over the probe, the probe is removed, and a standard chest tube (up to 36-French) is placed through the sheath. While decompression of simulated tension pneumothorax was achieved significantly more often using the Reactor™ as compared to standard 14-gauge needle decompression in a post-mortem swine model, performance of this device in a human model is lacking [12].

We sought to evaluate the efficacy of chest tube insertion using the Reactor™ device in comparison to the standard, open technique in a randomized, controlled study using human cadavers. We chose to evaluate novice users to provide external validity to providers of this type, who perform a significant proportion of chest tube procedures in the trauma setting. We hypothesized that tube thoracostomy using the Reactor™ would be performed with a shorter incision length, faster insertion time, superior tube placement, and greater subjective ease of use compared to the conventional open technique.

## Methods

### Study design and setting

We conducted a randomized, six-period, two-treatment, crossover study comparing the Reactor™ technique to the standard tube thoracostomy technique performed by novice users on human cadavers in two sessions in December 2017 and January 2018. Cadavers were supplied by the Maryland State Anatomy Board. Sharp Medical Products, LLC. supplied the Reactor™ devices and paid for the use of the cadavers, but was otherwise uninvolved with the study conduct, data collection, statistical analysis or publication of the results.

### Participants

We recruited first and second year medical students from the University of Maryland School of Medicine and national registered paramedics certified in the state of Maryland. Voluntary participation was sought through e-mail advertisements, flyers, and announcements made in medical school and emergency medical services interest groups. Subjects were required to have never performed chest tube placement on a live patient but could have performed prior procedures using manikin, animal or human cadaver models.

### Randomization and treatment sequence assignment

Each study participant was assigned to perform a total of six chest tube insertions, three using the Reactor™ technique and three using the standard technique, completed in a pre-specified alternating order. Since the subjects were all novice users, we anticipated that procedural competency could increase during the study from the first to the sixth chest tube insertion. To achieve an equal total number of insertions using each of the two techniques and an equal probability of each technique being performed for the first through sixth procedure, a study-order matrix was designed (Table 1). Using a computer-generated random number sequence, subjects were randomly assigned to a particular sequence of chest tube insertions. Unblinded assignment was performed by one group of study investigators who later performed the unblinded portion of data collection. Treatment allocation was blinded, however, to those investigators that later ascertained the primary outcome.

**Table 1:** Study-Order sequence assignment matrix

	Subject															
Method	(1)	(2)	(3)	(4)	(5)	(6)	(7)	(8)	(9)	(10)	(11)	(12)	(13)	(14)	(15)	(16)
R	4	5	6	**1**	2	3	4	5	6	**1**	2	3	4	5	6	**1**
S	5	6	**1**	2	3	4	5	6	**1**	2	3	4	5	6	**1**	2
R	6	**1**	2	3	4	5	6	**1**	2	3	4	5	6	**1**	2	3
S	**1**	2	3	4	5	6	**1**	2	3	4	5	6	**1**	2	3	4
R	2	3	4	5	6	**1**	2	3	4	5	6	**1**	2	3	4	5
S	3	4	5	6	**1**	2	3	4	5	6	**1**	2	3	4	5	6

*R* Reactor technique, *S* Standard technique; (Numbers) represent individual study participants; Numbers below each labelled study participant in each column represent the order of chest tube insertion (**1 = first**, 6 = sixth)

### Cadaver model

**Fig. 2:**
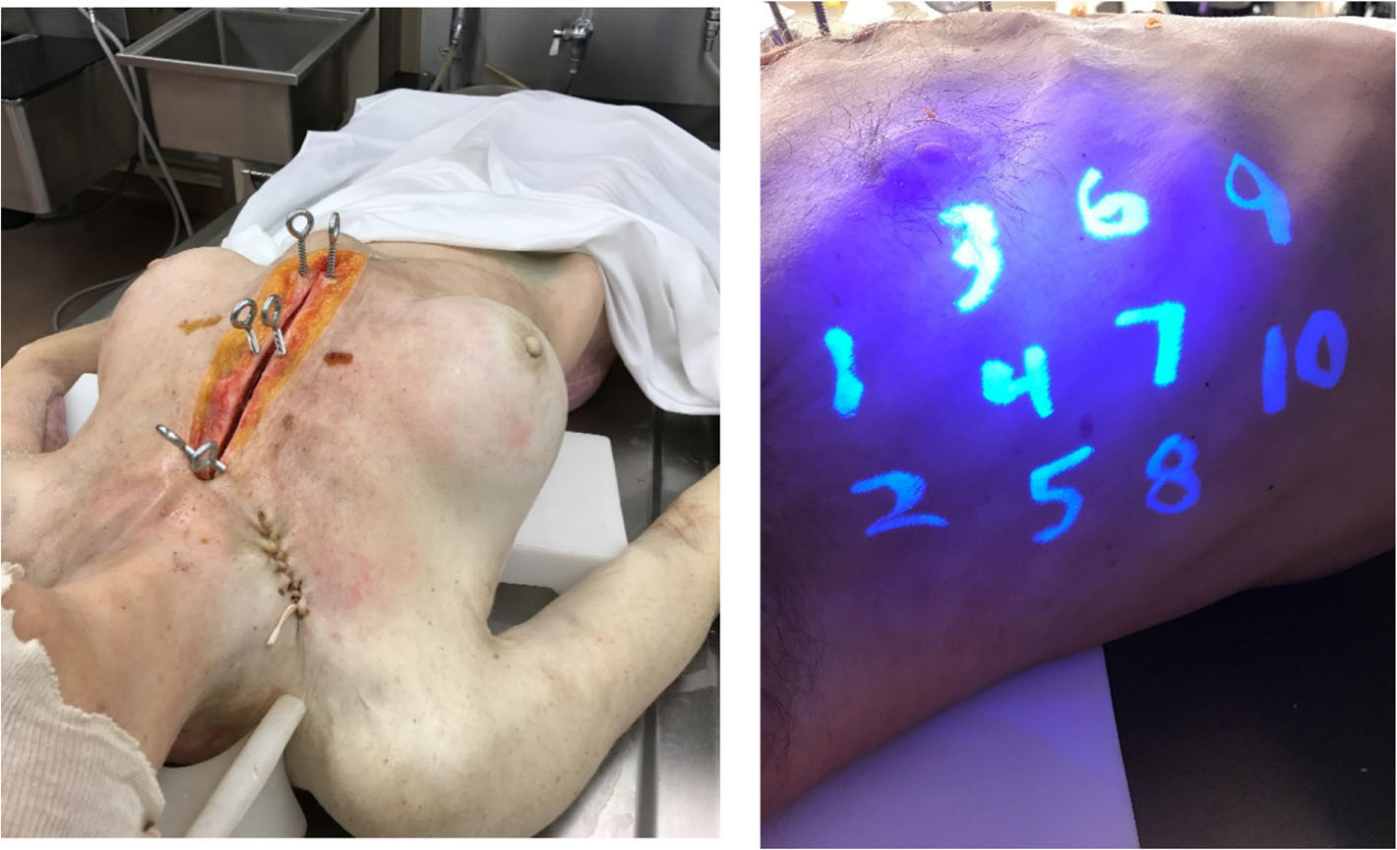
Human Cadaver Model. **a** (left) demonstrates the setup of the median sternotomy used to evaluate tube placement. **b** (right) displays the numbering system used to mark the pre-specified locations to chest tube insertion

Tube thoracostomy was performed on fresh, nonembalmed human cadavers, stored at 4 °C and allowed to warm to room temperature prior to use. Prior to study initiation, a median sternotomy was performed and both medial pleural borders were widely opened to allow for visual and tactile assessment of each hemithorax. Two to three pairs of two-inch eye screws were inserted into each half of the sternum and secured together with rubber bands to reapproximate the shape of the thoracic cavity (Fig. 2a). The cadavers were positioned supine with corpse elevators beneath both scapula and with the arms abducted to 90 degrees.

To simulate realistic conditions in which novice users may not correctly identify the ideal insertion site for chest tube placement (anterior-mid axillary line, 4th or 5th intercostal space) [13, 14], we divided each hemithorax into ten potential insertion sites (corresponding roughly to regions between the anterior and posterior axillary lines and from the 3rd to 6th intercostal spaces). Each location was labeled in colored marker visible only under ultraviolet-light (Fig. 2b). Prior to randomization of the study participants, we assigned each pre-specified chest tube procedure in the study-order matrix (each cell in Table 1) to a unique insertion site on one hemithorax of one of the cadavers. For each hemithorax of each cadaver, a separate order of insertion sites was created using a computerized random number sequence from 1 to 10. For each study subject, starting with their first procedure the location of each tube insertion was assigned to a site on a cadaver based on the previously generated sequence for that cadavers’ hemithorax. For simplicity, the first procedure for each participant was always performed on the right hemithorax, with subsequent procedures alternating sides. Once all ten sites on a given hemithorax for a given cadaver were assigned, the following assignment proceeded to the next available cadaver, maintaining the order of laterality, using the unique random sequence of insertion sites linked to that cadaver’s hemithorax. Each insertion site was unique to one chest tube procedure; no locations were used more than once.

Unblinded study investigators utilized an ultraviolet flashlight to identify and drape the appropriate insertion location with surgical towels prior to each chest tube insertion. The combination of draping and ultraviolet-only visible marking was intended to blind the participants to the exact location on the cadaver at which they were performing each tube insertion.

### Interventions

All subjects received a protocolized 2-h training session prior to randomization. Instructional videos and concomitant narration of the standard and Reactor™ techniques were shown and the participants completed one chest tube insertion using each technique with real-time teaching by the study investigators. These procedures were performed on cadavers specifically used for training and not later utilized for the study implementation.

All chest tube insertions were performed using a 10 or 11-blade scalpel and a 32-French chest tube (Atrium Medical Corporation, Hudson, NH). Subjects were instructed to aim for placement in the posterior apex of the pleural cavity, corresponding to the customary desired position in the setting of undifferentiated thoracic trauma, and to avoid the neurovascular bundle by inserting the tube just over the rib.

For the standard tube thoracostomy technique, one 5.5-in. curved Kelly clamp and one 8-in. curved Kelly clamp were provided. Subjects were instructed in the steps of the standard technique as follows: 1) make a ~ 3 cm anteroposterior oriented incision through the skin, 2) using the 5.5-in. curved Kelly clamp blunt dissect through the subcutaneous tissue and enter the pleural space, 3) enlarge the pleurotomy in the anteroposterior direction by spreading the jaws of the clamp, 4) insert one finger into the pleural space and palpate the positioning deep to the ribs within the pleural cavity, 5) insert the chest tube using either the 8-in. curved Kelly clamp or one finger to guide the tip of the tube into the desired location.

For the Reactor™ technique, only the endoscopic device was provided in addition to the scalpel and chest tube. Subjects were instructed in the steps of the Reactor™ technique as follows: 1) make a ~ 2 cm anteroposterior oriented incision through the skin, 2) place the tip of the device into the incision and, applying constant pressure, repeatedly depress the trigger mechanism to dissect through the subcutaneous tissue and enter the pleural space, 3) slide the sheath over the device barrel into the pleural space and then remove the device barrel, 4) insert the chest tube through the sheath, directing the angle/location as desired by manipulating the sheath, 5) remove the sheath over the chest tube. If the barrel or sheath of the device were unable to smoothly pass through the skin incision, participants were instructed to elongate the skin incision using the scalpel.

Procedures were observed by unblinded study investigators to ensure compliance with the assigned technique. Both techniques were considered complete when the subject indicated they were satisfied with the tube placement. The tube was not sutured to the skin nor connected to a negative pressure drainage system. After performing each two chest tube procedures, one on either side of the cadaver, the subjects stepped out of the lab while blinded study data collection was performed by a separate team of assessors. Following the blinded assessments, subjects returned and performed two more insertions, repeating these steps for a total of six insertions per participant.

### Data collection and outcome assessment

The primary outcome measure was successful tube placement, defined as a chest tube meeting all the following criteria: 1) distal tip and at least one side hole within the pleural space, 2) not terminating in the major fissure, 3) not kinked. All the side holes were not required to be within the pleural space to satisfy criteria 1), however the tube could not pass through the pleural space and then out of the pleural space (e.g. through the diaphragm) for criteria 1) to be satisfied. If a portion of the tube passed through the major fissure but at least one side hole at the distal aspect was outside of the fissure, the tube was considered to satisfy criteria 2). A kink was defined as an abrupt change in direction that resulted in a ≥ 90-degree angle deviation in tube shape.

Study investigators blinded to insertion technique measured and recorded tube position after every 2 procedures (one on each hemithorax of the cadaver), with study participants not present. The rubber bands connecting the eye screws placed in the cadaver’s sternum were removed and the sternotomy was retracted, allowing access to bilateral pleural cavities. Tube position was determined by manual palpation of the course of the tube from entrance into the pleural space to its distal tip, with the aid of visualization if needed. After position was determined, the tubes were removed and the sternum was reapproximated. This procedure was repeated for every pair of tube insertions.

Secondary outcomes included: skin incision length (millimeters), measured using a ruler by the blinded investigators, procedure time from start of incision to satisfactory tube placement (seconds), measured with a stopwatch by the unblinded study investigators, and subjective level of difficulty of each tube insertion (1–10 on a Likert scale, 1 being easiest, 10 being most difficult) rated by the participants after each procedure and recorded by the unblinded study investigators. A nine-question survey was distributed upon completion of the study to evaluate participants’ impressions of the Reactor™ compared to the conventional open technique. Participant type (medical student or paramedic) and prior experience with simulated chest tube placement was also documented.

### Sample size calculation and statistical analysis

The study was initially powered to satisfy the sample size requirement for a primary outcome of incision size. The hypothesis was originally that an incision size of less than 50% (2 cm vs. 4 cm) would be observed for chest tube insertion with the Reactor™. Assuming 80% power with an alpha level of 0.05 and standard deviation of 1 cm, only 8 participants would be required. However, prior to study initiation, a more clinically relevant outcome was considered. For the outcome of proportion of correct chest tube placements, a two-sample paired-proportions test was used, assuming 80% power and an alpha level of 0.05. With a hypothesized success rate of 80% correct placement with the standard open technique, a sample size of 15 subjects was calculated.

Descriptive statistics were performed with use of the Wilcoxon rank sum test for non-parametrically distributed continuous data and the Student’s t-test for parametrically distributed continuous data. Histograms, q-q plots, and the Shapiro-Wilk test were used to assess normality of continuous data. The Fisher’s exact test was used to analyze categorical data given the relatively small sample size. All tests were two-tailed, and a relatively stringent *P* value of < 0.005 was considered statistically significant [15, 16]. All analyses were performed using Stata version 15.1 (StataCorp, College Station, TX, USA) and GraphPad Prism version 7.0d for Mac (GraphPad Software, La Jolla, CA, USA).

## Results

Sixteen subjects participated in the study, 7 (44%) were first- or second-year medical students and 9 (56%) were nationally registered paramedics. Twelve (75%) subjects had no prior experience with simulated chest tube placement and 4 (25%) had performed one or more simulated procedures. Fourteen (87.5%) subjects performed 6 chest tube insertions (3 Reactor™, 3 standard), while 2 subjects (12.5%) only completed 4 chest tube procedures (2 Reactor™, 2 standard) due to limited anatomy lab availability. In total, 92 chest tube insertions (46 Reactor™, 46 standard) were performed by participants, all using the pre-assigned order/technique.

Unadjusted outcomes are presented in Table 2. The primary outcome, frequency of successful tube placement, occurred significantly more often using the standard technique compared with the Reactor™ (73.9% vs. 41.3%, *p* = 0.0029). The individual components of successful tube placement favored the standard technique but were not significantly different by themselves between groups. In total, 9 chest tubes (2 standard, 7 Reactor™) were not placed within the pleural space. The 2 extra-pleural tubes in the standard group were entirely within the subcutaneous space, 5 of the extra-pleural Reactor™ tubes were fully subcutaneous and 2 of the extra-pleural Reactor™ tubes entered the pleural space but then coursed through the diaphragm into the liver. The Reactor™ was associated with statistically significantly faster median insertion time (~ 30 s) and shorter incision length (~ 4 mm) compared with the standard technique. Study participants’ rating of the overall difficulty of tube placement was not different between groups.

**Table 2:** Unadjusted outcomes

Outcome	Standard Technique *N* = 46	Reactor™ Technique *N* = 46	*P*-value
Successful tube placement, n (%)	34 (73.9)	19 (41.3)	0.0029
Correctly placed in pleural space, n (%)	44 (95.6)	39 (84.8)	0.158
Kinked tube, n (%)	8 (17.4)	14 (30.4)	0.051
Placed in fissure, n (%)	3 (6.5)	7 (15.2)	0.075
Insertion time (sec) Median (IQR)	76.9 (55.3–106.9)	47.3 (38–63.1)	< 0.0001
Incision length (mm) Median (IQR)	32 (26–40)	28 (23–30)	0.0034
Perceived difficulty (1–10 scale; 10 most difficult) Mean (SD)	4.7 (1.9)	3.8 (1.9)	0.024

*IQR* interquartile range, *SD* standard deviation

**Fig. 3:**
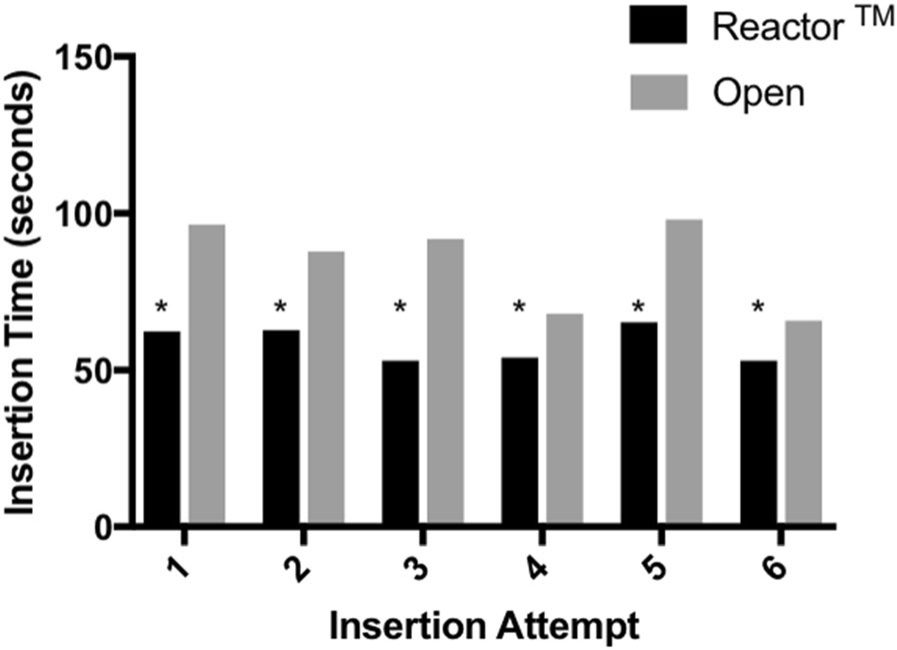
Insertion Time for the Reactor™ vs. Standard Technique Over the Course of Successive Attempts. *P < 0.005

**Fig. 4:**
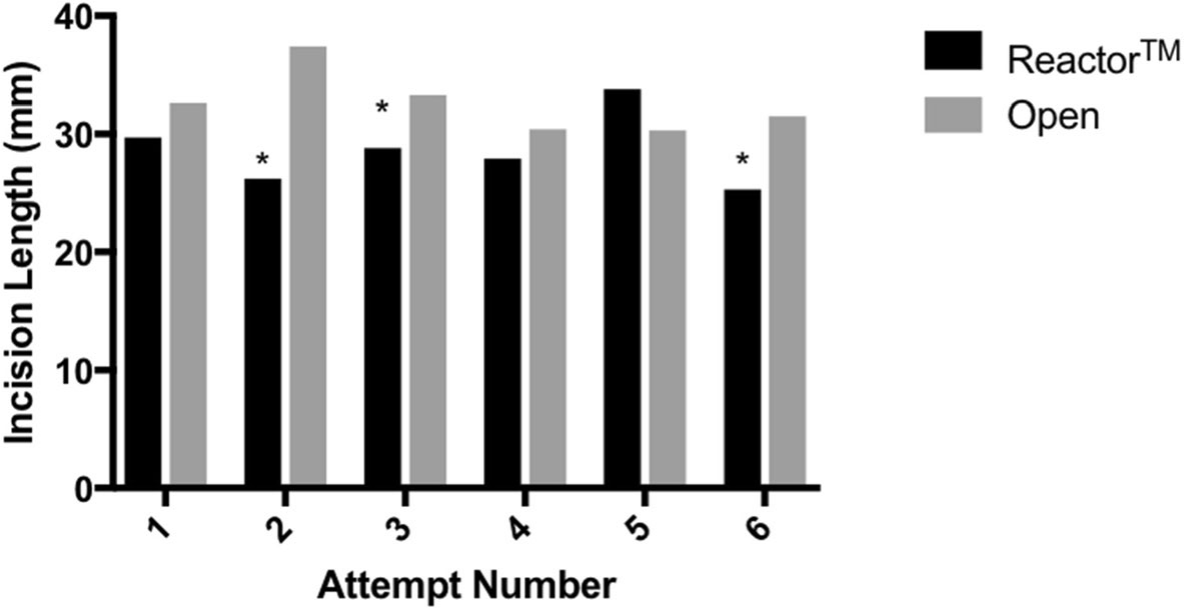
Mean Incision Length for the Reactor™ vs. Standard Technique Over the Course of Successive Attempts. *P < 0.005

**Fig. 5:**
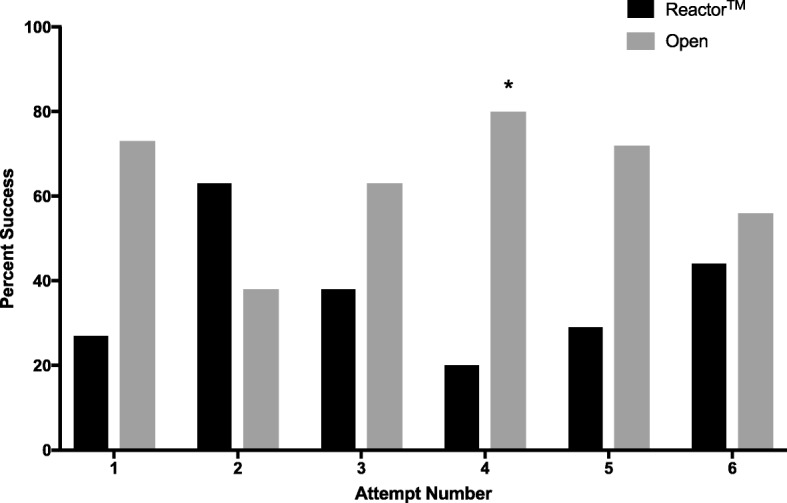
Percentage of Successful Tube Placements for the Reactor™ vs. Standard Technique Over the Course of Successive Attempts. *P < 0.005

Over successive attempts, insertion time remained significantly faster in the Reactor™ group compared to the open technique (Fig. 3), while difference in incision length was inconsistently significantly shorter with the Reactor™ device (Fig. 4). The frequency of successful tube placement was also inconsistently greater in the standard technique group over repeated attempts (Fig. 5).

**Fig. 6:**
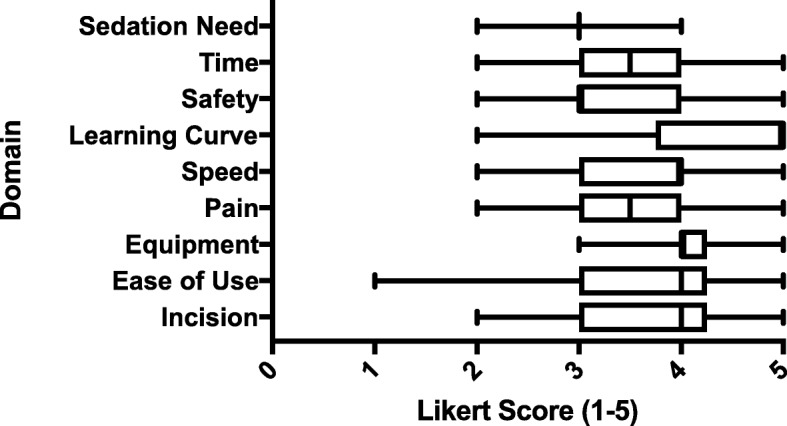
Results of Post-participation Survey. A Likert scale of 1 (strongly disagree) to 5 (strongly agree) was used to assess various subjective parameters comparing the Reactor™ to the standard technique. *Survey key:* Compared to the open insertion technique, the Reactor™ device: 1. (*Incision*) Is associated with a smaller incision? 2. (*Ease of Use*) Is easier to use? 3. (*Equipment*) Requires less equipment? 4. (*Pain*) Is likely to cause LESS pain for the patient? 5. (*Speed*) Allows for a chest tube to be inserted more rapidly? 6. (*Learning Curve*) Is NOT as difficult to learn? 7. (*Safety*) Improves clinician safety (i.e., needle sticks, injuries, etc.)? 8. (*Time*) Requires LESS time to insert a chest tube? 9. (*Sedation Need*) Is likely to require LESS sedation and analgesia?

Results from the end-of-study survey are shown in Fig. 6; the Reactor™ received generally favorable scores for all parameters.

## Discussion

In this randomized, assessor-blinded, crossover cadaver study among novice participants, use of the Reactor™ device for chest tube insertion resulted in significantly less frequent successful tube placement, but was associated with a faster insertion time and shorter incision length compared with the standard, open technique. Participants rated the ease of use of Reactor™ more favorably compared to the standard open technique, but using a stringent statistical cut-off (i.e., a *P* value < 0.005), these results were not statistically significant.

For providers in prehospital, military, or intensive care settings, pneumothorax and hemothorax are potentially lethal conditions and must be treated promptly [2, 9, 17]. Unfortunately, both needle decompression and tube thoracostomy are infrequently applied by many non-surgeons in these environments, and a high rate of failure has been observed with the classic technique of needle decompression for tension pneumothorax [18–20]. A compact, efficacious, easy-to-use device that requires less technical skill may be appealing for these scenarios. One prior animal study has described the use of a laparoscopic-type trocar for decompression of tension pneumothorax and found the technique to be more effective than standard needle decompression [21]. Furthermore, Kuckelman et al. showed that the Reactor™ was significantly more successful at achieving rapid and complete resolution of tension pneumothorax compared to needle decompression (88% vs. 44%, *P* < 0.001) in a post-mortem swine model. Laparoscopic-type trocar procedures have been reported in two human case series for the treatment of simple pneumothoraces in which a chest tube is subsequently inserted through the trocar [4, 5]. Nevertheless, tube thoracostomy using a trocar-type device or the Reactor™ has not yet been compared with the standard, open technique.

The Reactor™ is a novel device with several purported design advantages for the performance of tube thoracostomy. The device uses a rotating and retracting blade that “carves” rather than punctures the subcutaneous layer, potentially resulting in less tissue damage. The oval shaft is designed to fit between ribs, potentially avoiding intercostal vascular or nerve injuries. Medium to large chest tubes can be inserted with potentially shorter incisions due to a “tunneling” effect created by the device during subcutaneous dissection. Importantly, the device has the potential to decrease the incision length required for tube insertion and can be performed rapidly, thereby potentially resulting in less patient discomfort. These design features may be especially advantageous for less experienced providers tasked with performing chest tube insertion in a variety of settings.

In this study, novice proceduralists confirmed some of the design advantages (shorter incision length, faster insertion time) of the Reactor™ in a cadaver model. Given the high prevalence of pain, pulmonary complications, and morbidity associated with both blunt and penetrating thoracic trauma, novel techniques that can lessen the discomfort associated with tube thoracostomy are highly desirable. The need for local and parenteral analgesia during tube thoracostomy placement—including opioids—is substantial [22]. In order to truly assess pain and analgesic needs with the Reactor™ compared to the standard technique and to evaluate the clinical significance of the small differences in insertion time and incision length we found, a human study would be needed using validated measures for assessment of pain.

By contrast, the primary outcome in this study—appropriate chest tube placement—was less frequently achieved in the Reactor™ group compared to the standard technique. The potentially life-threatening nature of the conditions treated by tube thoracostomy and the significant harm that can result from malpositioned chest tubes necessitates that any new procedural technique has at least equivalent safety and efficacy compared to the standard, open procedure [3, 10]. Our results suggest this may not be the case with the Reactor™. Evaluation of the occurrence of the individual criteria for successful tube placement suggests the difference between groups was primarily due to more frequent kinking and placement in the fissure with the Reactor™ technique. This could be because chest tubes placed using the Reactor™ are inserted into the thoracic cavity through the short introducer sheath, which limits tactile feedback on the initial path of the tube compared to the open technique, where a finger can be inserted into the chest to guide the tube in a particular direction. Extra-pleural tube placement also occurred in 7/46 (15%) Reactor™ procedures compared to 2/46 (4%) insertions using the standard technique. We purposely manipulated site selection to include locations predisposed to more frequent complications to simulate potential application by novice users, since prior data has shown an ~ 5% rate of extra-pleural tube placement and as high as a 60% rate of placement outside of the ideal location (4th or 5th intercostal space) when chest tubes are placed by trainees under emergent scenarios [13, 14]. A notable limitation of the Reactor™ technique is that it does not allow for confirmation of entrance into the pleural cavity through insertion of a finger into the chest and palpation of the inner surface of the ribs. Though our study was not powered to show a significant difference in the frequency of extra-pleural tube placement, the overall results suggest that further research is needed to critically evaluate the safety of the Reactor™ device.

Our study has several strengths, including the randomized, crossover design, blinded primary outcome assessment, and recruitment of a population of novice users from different backgrounds. Conversely, our results may not be generalizable to more technically experienced providers and are inherently limited by the nature of the human cadaver model. We observed a higher than expected frequency of tube misplacement among both techniques, emphasizing the significant learning curve for chest tube placement. Tube thoracostomy is an advanced skill that requires practice, experience, and proficiency, regardless of the type of insertion technique.

## Conclusions

In summary, this randomized, assessor-blinded, crossover cadaver study comparing the Reactor™ to standard tube thoracostomy technique in novice participants showed chest tube insertion was more frequently successful with the standard, open technique but slightly faster and associated with a shorter incision length using the Reactor™ device. Further research is needed to scrutinize the safety and efficacy of the Reactor™ device and investigate whether it has clinically significant benefits over the standard technique with regard to insertion time, incision length, and procedural discomfort when used in patients.

## Abbreviation

FDA: Food and Drug Administration
